# Supplementary figures and images for: BioFET-SIM Web Interface: Implementation and Two Applications
Source: PLoS One. 2012 Oct 8;7(10):e45379. doi: 10.1371/journal.pone.0045379 (PMC3466287; doi:10.1371/journal.pone.0045379)

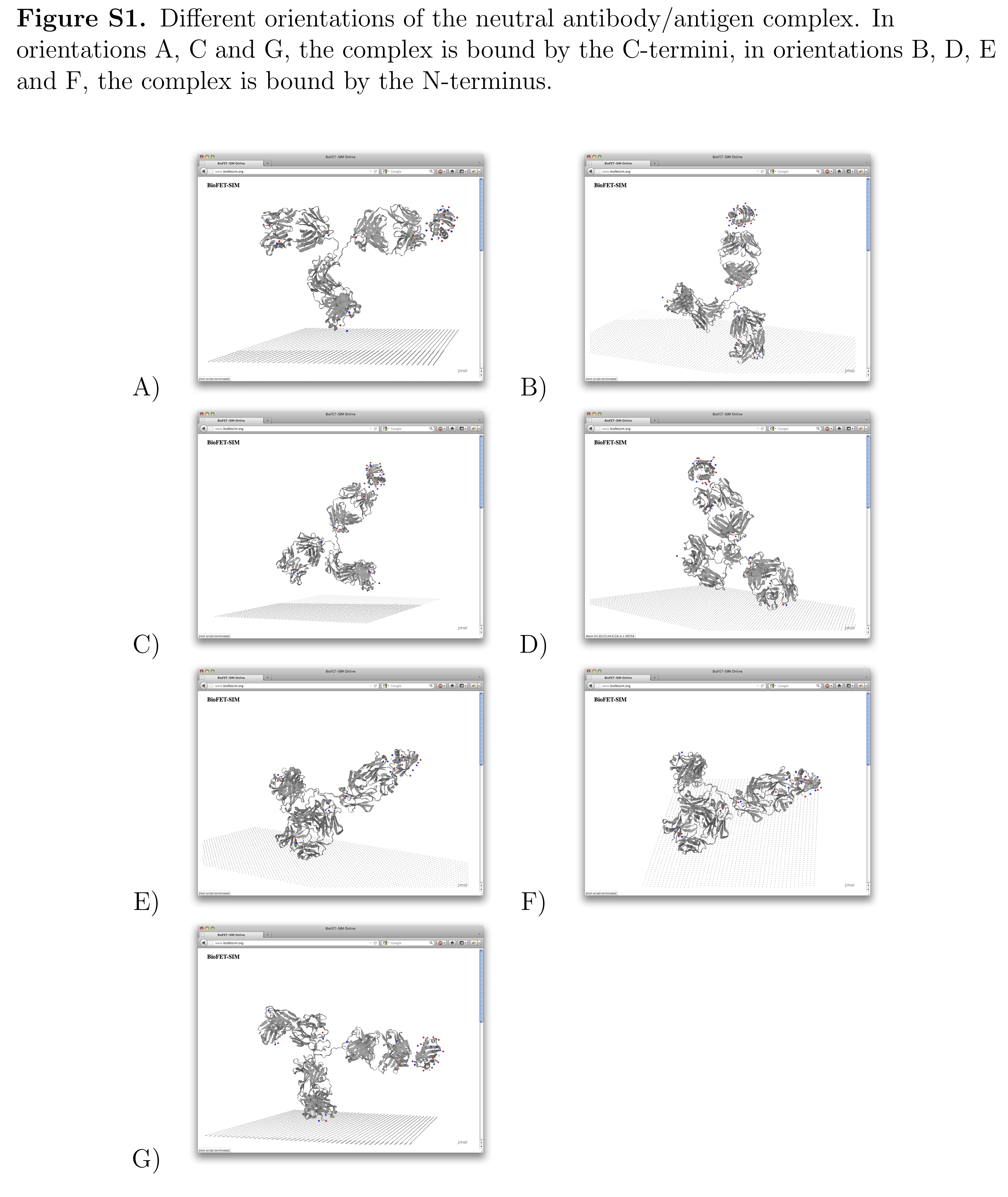

Supplement: Figure S1 — Different orientations of the neutral antibody/antigen complex. In orientations A, C and G, the complex is bound by the C-termini, in orientations B, D, E and F, the complex is bound by the N-terminus. (TIF) [file pone.0045379.s001.tif]

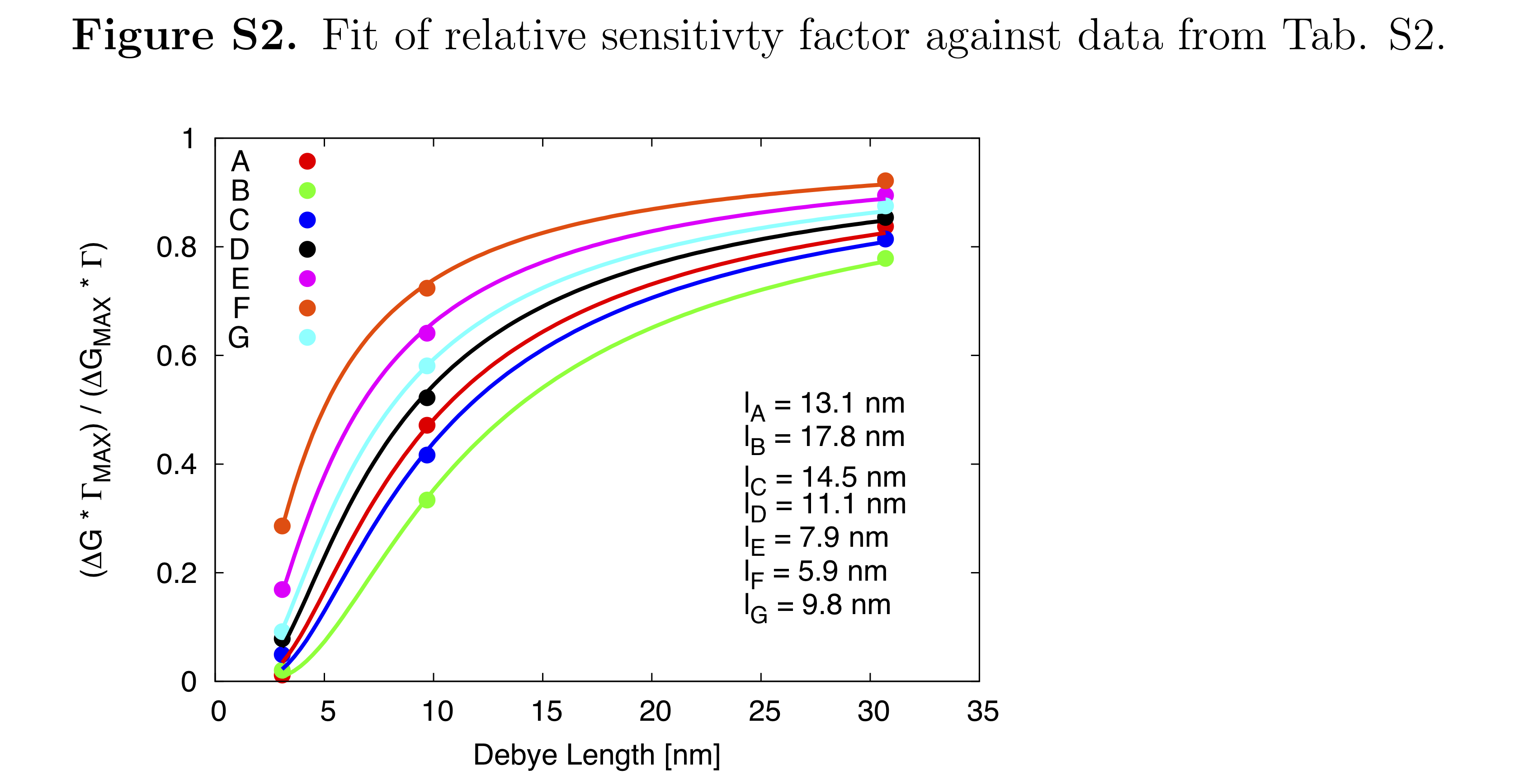

Supplement: Figure S2 — Relative sensitivity factor. Fit of relative sensitivity factor against data from Tab. S2. (TIF) [file pone.0045379.s002.tif]

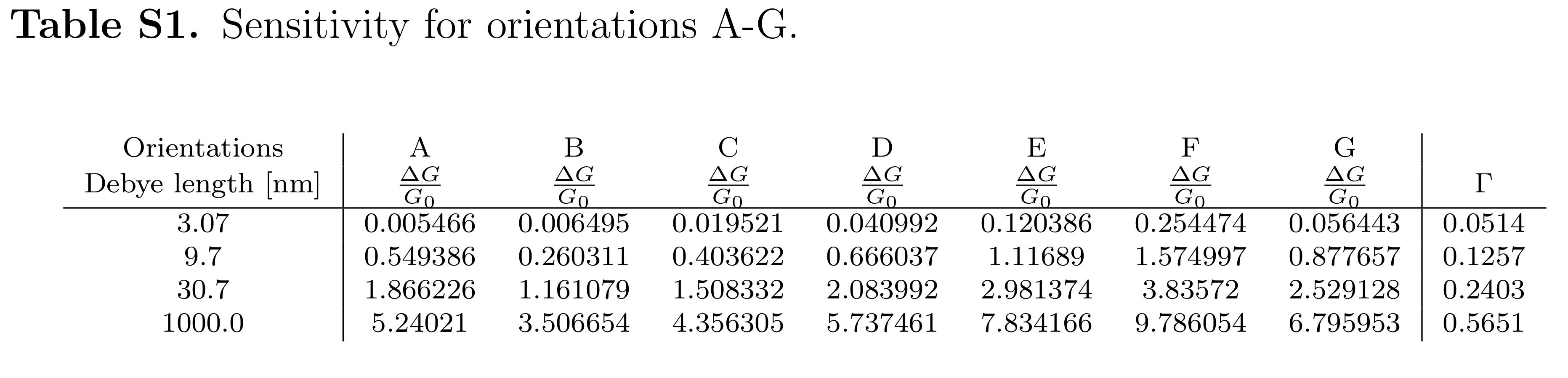

Supplement: Table S1 — Antibody study. Sensitivity for orientations A-G. (TIF) [file pone.0045379.s003.tif]

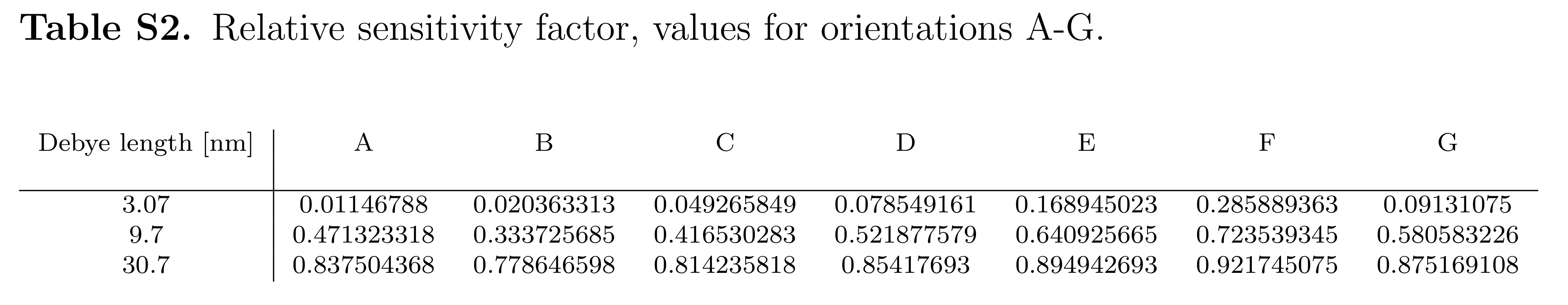

Supplement: Table S2 — Antibody study. Relative sensitivity factor for orientations A-G. (TIF) [file pone.0045379.s004.tif]
