# Supplementary material for: BioFET-SIM Web Interface: Implementation and Two Applications
Source: PLoS One. 2012 Oct 8;7(10):e45379. doi: 10.1371/journal.pone.0045379 (PMC3466287; doi:10.1371/journal.pone.0045379)
Supplement: Text S1 — Supporting material. (PDF) [file pone.0045379.s005.pdf]

Electronic Supplementary Information

BioFET-SIM Web Interface: Implementation and Two Applications

Martin R. Hediger, Jan H. Jensen, Luca De Vico

S1.1 Derivation of Relative Nanowire Sensitivity Factor

According to our model, the sensitivity of a generic p-type nanowire, in the single charge approximation, can be expressed as

$$\frac{\Delta G}{G_0} = -\frac{2}{R\, e\, p_0} \Gamma\left(\Gamma_l\, \sigma_b + \sigma_s\right) \tag{S1}$$

and the base conductance G_0 can be expressed as

$$G_0 = \frac{\pi\, R^2\, e\, p_0\, \mu}{L} \tag{S2}$$

In these equations e is the elementary charge, R is the radius of the nanowire and p_0 the hole density, μ is the charge carrier mobility. Γ and Γ_l are dimensionless functions quantifying the actual sensitivity of the nanowire and they depend, among other parameters, on the distance l of the sensed charge from the nanowire surface and the buffer Debye length λ_D [1]. When using a multiple charge model, we interpret l as the average distance of the sensed charges from the nanowire surface.

If the physical and geometrical properties of the nanowire are fixed, G_0 is constant. If we only consider the sensing of charges immersed in the buffer (i.e. let $\sigma_s = 0$), Eq. S1 can be simplified and it is possible to express the change in conductivity as

$$\Delta G = K\Gamma\, \Gamma_l\, \sigma_b \tag{S3}$$

where K collects all constant values. Using the expression for Γ_l

$$\Gamma_l = 2\frac{R}{R+l}\left(1 + \sqrt{\frac{R}{R+l}}\exp(l/\lambda_D)\right)^{-1} \tag{S4}$$

we can now define a value for the buffer Debye length at maximum dilution, λ_D^{max}, and express the maximum change in conductivity at this value as

$$\Delta G^{max} = K\Gamma^{max}\, \Gamma_l^{max}\, \sigma_b. \tag{S5}$$

When considering a highly diluted buffer, i.e. $\lambda_D^{max} \gg l$, we can express Γ_l^{max} as

$$\Gamma_l^{max} \simeq 2\frac{R}{R+l}\left(1+\sqrt{\frac{R}{R+l}}\right)^{-1}. \tag{S6}$$

The ratio between the change in conductivity at a given Debye length and the maximum possible value becomes

$$\frac{\Delta G}{\Delta G^{max}} = \frac{K\Gamma\,\Gamma_l\,\sigma_b}{K\Gamma^{max}\,\Gamma_l^{max}\,\sigma_b} = \frac{\Gamma\,\Gamma_l}{\Gamma^{max}\,\Gamma_l^{max}} \tag{S7}$$

and after reordering it is possible to obtain

$$\frac{\Delta G\,\Gamma^{max}}{\Delta G^{max}\,\Gamma} = \frac{\Gamma_l}{\Gamma_l^{max}}. \tag{S8}$$

After inserting the explicit expressions, we obtain

$$\frac{\Gamma_l}{\Gamma_l^{max}} = \frac{2\frac{R}{R+l}\left(1+\sqrt{\frac{R}{R+l}}\exp(l/\lambda_D)\right)^{-1}}{2\frac{R}{R+l}\left(1+\sqrt{\frac{R}{R+l}}\right)^{-1}} \tag{S9}$$

where we define Γ_l/Γ_l^{max} as the *relative sensitivity factor.*

Using Eq. 2 from previously published work [1], it is possible to compute the values of Γ for different Debye lengths, including Γ^{max} for $\lambda_D^{max} = 1000$nm. Using BioFET-SIM it is possible to obtain the value of ΔG (and ΔG^{max}) simply by multiplying $\frac{\Delta G}{G_0}$ with G_0. It is then possible to plot the l.h.s. of Eq. S8 for different values of λ_D. This plot can be fitted to the r.h.s. of Eq. S9 where l is the fitting paramenter. From a series of measures at different Debye lengths, it is then possible to obtain the average distance of the sensed charge from the nanowire surface.

S1.2 Expression for Γ

For simplification of notation, in the expression for Γ, we use the thickness $t := R_{NW} + l_{ox}$. Then, Γ is given by

$$\Gamma = \frac{\epsilon_1 \cdot K_0\left(\frac{t}{\lambda_D}\right)\frac{\lambda_D}{\lambda_{TF}} \cdot I_1\left(\frac{R_{NW}}{\lambda_{TF}}\right)}{\left[K_0\left(\frac{t}{\lambda_D}\right)\cdot\frac{\lambda_D}{t} + ln\left(\frac{t}{R_{NW}}\right)\cdot K_1\left(\frac{t}{\lambda_D}\right)\frac{\epsilon_3}{\epsilon_2}\right]\epsilon_1\frac{R_{NW}}{\lambda_{TF}}\cdot I_1\left(\frac{R_{NW}}{\lambda_{TF}}\right) + \epsilon_3\cdot K_1\left(\frac{t}{\lambda_D}\right)\cdot I_0\left(\frac{R_{NW}}{\lambda_{TF}}\right)} \tag{S10}$$

In Eq. S10, I_0, I_1, K_0 and K_1 are the modified Bessel functions of first and second kind, respectively [1].

S1.3 Antibody and Antigen Preparation

A suitable complex structure of a generic antibody and an antigen used by Vacic et al. [2] was prepared. Only few full antibody structures have been resolved. We used the structure of an intact IgG2a monoclonal antibody, ascension code 1IGT [3]. For the antigen we used the structure of the SEA domain of human mucin 1, with ascension code MUC1 [4].

The antigen structure was rigidly docked to the antigen-binding site of the antibody using AutoDock [5] and visually checked with the program PyMOL [6] for a reasonable docking. The scope of this docking was only to obtain a feasible complex structure.

Since we were interested in the sensing of only the antigen, the antibody structure had to be made as neutral as possible. In order to make the antibody as neutral as possible, all positions in the antibody sequence were mutated to glycine using PyMOL. When BioFET-SIM computes the charges of a protein, a positive charge is assigned to the N-terminus and a negative charge to the C-terminus, depending on the corresponding pK_a values calculated by PROPKA [7]. In order to counter balance the charges of the termini, in each of the four chains of the antibody we mutated the residue at the N-termini to aspartate and the residue at the C-termini to arginine.

S1.4 Antigen Sensing

The BioFET-SIM parameters were set as in Tab. 1. With these parameters we obtain $G_0 = 279.0$ nS.

The different orientations of the neutral antibody/antigen model complex which were tested are shown in Fig. S1. A biofunctionalization layer of 0.5 and 1.0nm was added for C- and N-terminus binding to the nanowire surface, respectively. We considered a pH of 7.4 and kept the number of proteins fixed to 4000 protein units. The BioFET-SIM results for all orientations A-G at the values of Debye length employed by Vacic et al. (3.07, 9.7 and 30.7nm) and $\lambda_D^{max} = 1000$nm are reported in Tab. S1, together with the corresponding values for Γ. In Tab. S2, the derived values for the l.h.s. of Eq. S8 are reported. The data from Tab. S2 is plotted in Fig. S2, together with the fitting parameter.

Figure S1. Different orientations of the neutral antibody/antigen complex. In orientations A, C and G, the complex is bound by the C-termini, in orientations B, D, E and F, the complex is bound by the N-terminus.

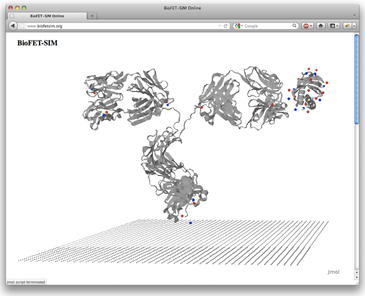

A)

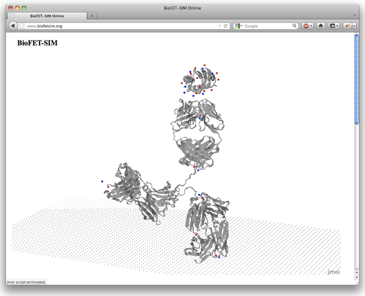

B)

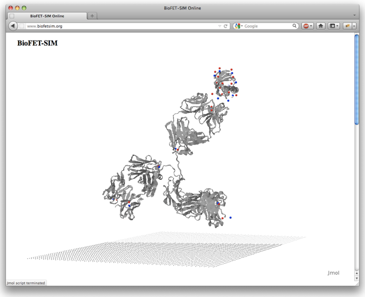

C)

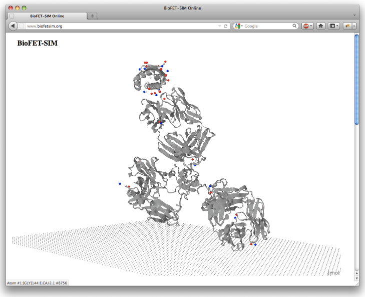

D)

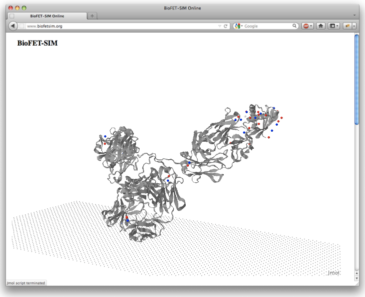

E)

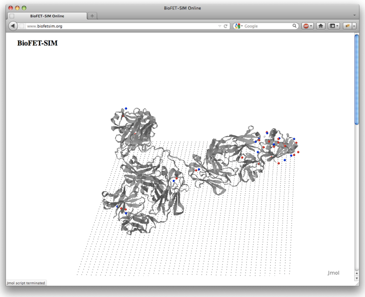

F)

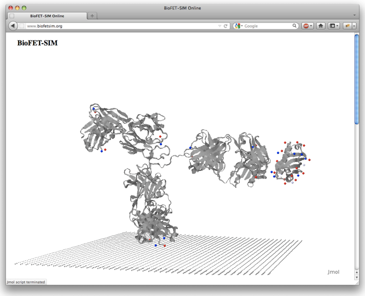

G)

Figure S2. Fit of relative sensitivty factor against data from Tab. S2.

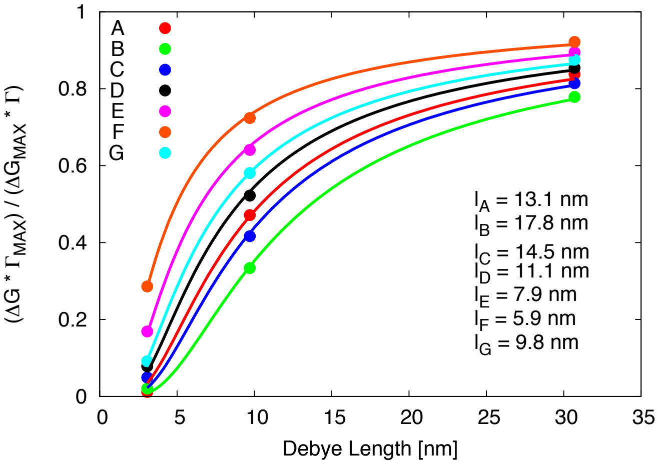

Table S1. Sensitivity for orientations A-G.

Orientations<br>Debye length [nm]	A<br>$\frac{\Delta G}{G_0}$	B<br>$\frac{\Delta G}{G_0}$	C<br>$\frac{\Delta G}{G_0}$	D<br>$\frac{\Delta G}{G_0}$	E<br>$\frac{\Delta G}{G_0}$	F<br>$\frac{\Delta G}{G_0}$	G<br>$\frac{\Delta G}{G_0}$	Γ
3.07	0.005466	0.006495	0.019521	0.040992	0.120386	0.254474	0.056443	0.0514
9.7	0.549386	0.260311	0.403622	0.666037	1.11689	1.574997	0.877657	0.1257
30.7	1.866226	1.161079	1.508332	2.083992	2.981374	3.83572	2.529128	0.2403
1000.0	5.24021	3.506654	4.356305	5.737461	7.834166	9.786054	6.795953	0.5651

Table S2. Relative sensitivity factor, values for orientations A-G.

Debye length [nm]	A	B	C	D	E	F	G
3.07	0.01146788	0.020363313	0.049265849	0.078549161	0.168945023	0.285889363	0.09131075
9.7	0.471323318	0.333725685	0.416530283	0.521877579	0.640925665	0.723539345	0.580583226
30.7	0.837504368	0.778646598	0.814235818	0.85417693	0.894942693	0.921745075	0.875169108

S1.5 Command Line Version of BioFET-SIM Usage

PDB Format Requirements:
The uploaded PDB file is required to contain the `MODEL`, `TER` and `END` tags. In addition, individual chains of the structure are required to contain the chain label (i.e. `A`, `B`, ...).

```
MODEL        1
ATOM      1  N   ASN A   2       0.209  -1.748  -0.613  1.00  0.00
...
TER
ATOM      1  N   HIS B   2      12.057   2.821   9.469  1.00  0.00
...
END
```

A number of instruction videos are available, the links to these are found on the interface website, www.biofetsim.org. The link to the command line version repository is found at the same URL.

System Requirements:
The command line version of the BioFET-SIM program requires the following applications and libraries to be installed on the local host.

- Python 2.5 or higher (not Python 3)
- Numpy and Scipy libraries installed

Basic usage is demonstrated by issuing

```
[user] $ python bio_run.py
BioFET-SIM usage:
$ python bio_run.py --calc <input.bfs>
or
$ python bio_run.py --set <param> <val> <input.bfs>
```

Starting a BioFET-SIM calculation using the input file "kk8add.bfs":

```
[user] $ python bio_run.py --calc kk8add.bfs
# BioFET-SIM Calculation
# Date of calculation: 2012-03-21 17:17:51
# Calculation target: kk8add
# pH: 7.4
# Comment: BFS Input generated by interface.

Adjustable Parameters:
L_d                     2.0
L_tf                   2.04
eps_1                  12.0
eps_2                   3.9
eps_3                  78.0
lay_bf                  1.0
lay_ox                  2.0
mu                     0.01
n_0                1.11e+24
num_prot               4000
num_qi                    6
nw_len               2000.0
nw_rad                 10.0
nw_type                   P
target               kk8add

Base Conductance [nS]: 279.352916413
Sensitivity:           0.115675065297
```

In the output, the labels have the following meaning (with the corresponding symbol given in Tab. 1, q_i given in Eq. 4): `L_d`: Debye screening length λ_D; `L_tf`: Thomas-Fermi screening length λ_{TF}; `eps_1`: Nanowire permittivity ϵ_1; `eps_2`: Oxide layer permittivity ϵ_2; `eps_3`: Solvent permittivity ϵ_3; `lay_bf`: Biolayer thickness l_b; `lay_ox`: Oxide layer thickness l_{ox}; `mu`: Charge carrier mobility μ; `n_0`: Charge

carrier density κ_0; `num_prot`: Number of biomolecules N; `num_qi`: Number of charged sites within each biomolecule q_i; `nw_len`: Nanowire length L_{NW}; `nw_rad`: Nanowire radius R_{NW}; `nw_type`: Nanowire doping type K; `target`: PDB identifier of the studied biomolecule.

A parameter can be adjusted by the ”–set” option, followed by the label for the corresponding parameter and the new value:

```
[user] $ python bio_run.py --set L_d 3.0 kk8add.bfs
Parameter adjusted.
```

References

1. De Vico L, Sørensen M, Iversen L, Rogers D, Sørensen B, et al. (2011) Quantifying signal changes in nano-wire based biosensors. Nanoscale 3: 706–717.

2. Vacic A, Criscione JM, Rajan NK, Stern E, Fahmy TM, et al. (2011) Determination of molecular configuration by debye length modulation. Journal of the American Chemical Society 133: 13886-13889.

3. Harris L, Larson S, Hasel K, McPherson A (1997) Refined structure of an intact igg2a monoclonal antibody. Biochemistry 36: 1581–1597.

4. Macao B, Johansson D, Hansson G, Härd T (2005) Autoproteolysis coupled to protein folding in the sea domain of the membrane-bound muc1 mucin. Nature structural & molecular biology 13: 71–76.

5. Goodsell D, Morris G, Olson A (1996) Automated docking of flexible ligands: applications of autodock. Journal of Molecular Recognition 9: 1–5.

6. The PyMOL Molecular Graphics System, Schrödinger, LLC (2010).

7. Olsson MHM, Sndergaard CR, Rostkowski M, Jensen JH (2011) Propka3: Consistent treatment of internal and surface residues in empirical pka predictions. Journal of Chemical Theory and Computation 7: 525-537.
